# The impact of the COVID-19 pandemic on mortality in people with dementia without COVID-19: a systematic review and meta-analysis

**DOI:** 10.1186/s12877-022-03602-6

**Published:** 2022-11-19

**Authors:** Michael Axenhus, Kristian Steen Frederiksen, Robin Ziyue Zhou, Gunhild Waldemar, Bengt Winblad

**Affiliations:** 1 Division of Neurogeriatrics, Department of Neurobiology, Care Sciences and Society, Karolinska Institutet, Solna, Sweden; 2 Theme Inflammation and Aging, Karolinska University hospital, Huddinge, Sweden; 3 Danish Dementia Research Centre, Department of Neurology, Rigshospitalet, University of Copenhagen, Copenhagen, Denmark; 4 Dept. of Clinical Medicine, University of Copenhagen, Copenhagen, Denmark

**Keywords:** Alzheimer’s disease, COVID-19, Dementia, Meta-analysis, Mortality, Systematic review

## Abstract

**Introduction:**

Significant mortality amongst vulnerable populations, such as people living with dementia, might go undetected during pandemic conditions due to refocus of care efforts. There is an urgent need to fully evaluate the pandemic impact on mortality amongst people living with dementia in order to facilitate future healthcare reforms and prevent deaths. The purpose of this study was to determine whether there was any significant difference in mortality amongst people with dementia without COVID-19 during the COVID-19 pandemic compared to previous years.

**Methods:**

A literature search was conducted in 5 databases. The relative risk ratio and confidence interval was used to estimate the change in mortality rates amongst people with dementia during the COVID-19 pandemic. The I^2^ value was used to assess heterogeneity, publication bias, and sensitivity analyses were performed.

**Results:**

Pooled analysis of 11 studies showed that mortality amongst people living with dementia was significantly increased during the COVID-19 pandemic for people with dementia without COVID-19. Mortality risk increased by 25% during the time period studied. Subgroup analysis was not performed due the low number of included studies.

**Conclusions:**

The results of this study suggest that people with dementia had a significant increased mortality during the pandemic even if they did not have COVID-19. People with dementia should participate in efforts that reduce general social spread and pandemic impact on healthcare system such as vaccinations, mask mandates, and testing. These results have clinical implications as preventing direct COVID-19 infection is not enough to adequately protect people living with dementia from increased mortality. Measures to limit social spread of infections and help support patients should also be a focus for clinicians. Further research should focus on the identification of mechanisms and other explanations for increased mortality as well as contributing factors such as living in care homes and differences between countries with various pandemic strategies.

**Supplementary Information:**

The online version contains supplementary material available at 10.1186/s12877-022-03602-6.

## Introduction

The Coronavirus disease 2019 (COVID-19) pandemic caused by the Severe acute respiratory syndrome-coronavirus 2 (SARS-CoV-2) virus has become a global public health crisis. In April of 2022, the World Health Organization reported almost 500 million confirmed cases and over 6 million deaths worldwide since the start of the pandemic [1]. Mounting evidence points towards certain patient groups being more vulnerable and exposed during the pandemic than others. In particular, elderly, immunocompromised, and people with comorbidities are patient groups that have shown a vulnerability to COVID-19 [2–4].

It is well known that frailty and old age are COVID-19 risk factors and that COVID-19 in the elderly lead to more debilitating symptoms [5–7]. Old age is also directly correlated with the severity of the disease [8]. The protection of the elderly population has been a challenge and countries have been subject to criticism because of failure to adequately safeguard their most vulnerable populations against the morbidity and mortality associated with COVID-19 [9–11]. Dementia, which is common amongst the elderly, is an important risk factor for developing severe illness in conjunction with COVID-19 and people with dementia have a higher risk of dying when infected [12].

Studies and reviews have so far focused on dementia as a risk factor for mortality due to COVID-19 and the direct effects of the COVID-19 pandemic on people living with dementia [12–14]. The many effects on a multitude of levels of a pandemic might cause disturbances to healthcare systems and put vulnerable patients at risk. The focus of care has been temporarily redirected towards acute care and away from long-term issues. To our knowledge, there are no systematic reviews or meta-analysis that examines mortality amongst people with dementia without COVID-19 during the pandemic, making our review the first to examine general mortality amongst people with dementia during the COVID-19 pandemic.

Non-communicable disease has been noted to be affected by the pandemic with fewer cancer diagnoses, increased mortality amongst patients with neurological diseases, and fewer planned surgeries [15–17]. Discussions have taken place about the increased mortality and infection rates in care homes, places were most are elderly, and many have dementia [18, 19]. Studies have reported worse quality of care and fewer doctor’s visits [20–23]. It is reasonable to suspect that people with dementia have been indirectly affected by the pandemic due to worse quality of care and refocus of healthcare.

The impact of the COVID-19 pandemic on the dementia population should therefore be thoroughly investigated as soon as possible. A higher mortality rate amongst people with dementia without COVID-19 would present new challenges for clinicians going forward as the prevention of direct infection might be insufficient to adequately protect people living with dementia during pandemic conditions.

This systemic review and meta-analysis was performed to explore whether dementia mortality amongst people with dementia without COVID-19 was increased during the COVID-19 pandemic through a pooled analysis of available literature. We also attempted to identify subgroups of vulnerable populations by analysis of home care settings, prevalence of mild and severe dementia, country specific epidemic situation, and prevention strategies.

## Materials and methods

This systematic review and meta-analysis was conducted according to the Preferred Reporting Items for Systematic Reviews and Meta-Analysis (PRISMA) guidelines [24]. The review protocol was registered at the International Prospective Register of Systematic reviews (PROSPERO, CRD42022316790). No institutional review board approval was required as all data have previously been published and no data used in this study can be used to identify individuals.

### Search strategy

A comprehensive search of several databases was conducted by one reviewer (RZZ) during March 2022 which included all the following databases: (1) PubMed; (2) EMBASE; (3) Web of Science; (4) CINAHL and (5) Cochrane Library. Results were retrieved from the inception of the databases to March 2022. The pandemic exposure was considered to have started at the 1st of January 2020 and onwards. Study data from before the 1st of January 2020 was considered to be without pandemic exposure. Studies published before 1st of January 2005 were excluded in order to emphasize more recent trends in mortality. The search was conducted using a combination of keywords including “COVID-19”, “dementia” and “mortality rate” and related terms. We also manually searched references of relevant literature to identify other eligible sources. Full details of the search strategy, including complete search strings, are provided in Supplemental Table 1.

### Eligibility criteria

Studies retrieved from the electronic databases were imported into the systematic review managing software Rayyan [25]. Deduplication was done in Rayyan using the algorithm imbedded in the software. All duplicates with less than 100% similarity were individually screened by a reviewer (MA) and excluded where appropriate. We relied on the methodology described by Woods in order to identify overlapping cohorts [26]. Inclusion criteria were: (1) the exposed groups were people with dementia during the COVID-19 pandemic without ongoing COVID-19; (2) The control group were people with dementia before the COVID-19 pandemic. (3) The primary outcomes were mortality rates or cause-specific death rate. (4) Results enabling relative risk (RR) or odds ratio (OR) calculation. Exclusion criteria: case reports, studies without control groups, expert opinions, editorial letters, and conference abstracts. An overview of inclusion and exclusion criteria are described in Table 1.

**Table 1:** Study designs and quality scoring according to the Newcastle Ottawa scale of non-randomized studies in meta-analysis and subsequent classification according to AHRQ standards

Study	Design	Bias rating Newcastle Ottawa			Quality
		Selection	Comparability	Outcome/Exposure	
Fedeli et al.	Case control	**	*	**	Moderate
Das-Munshi et al.	Cohort	***	*	***	High
Carey et al.	Cohort	***	*	***	High
Shiels et al.	Cohort	**	*	**	Moderate
Axenhus et al.	Cohort	**	*	**	Moderate
Reif et al.	Cohort	*	–	**	Low
Raknes et al.	Cohort	**	–	**	Low
Lee et al.	Cohort	**	*	**	Moderate
Strongman et al.	Cohort	***	*	***	High
Gilstrap et al.	Cohort	**	*	***	Moderate
Grande et al.	Cohort	**	–	**	Low

All articles were screened on title and abstract level by two reviewers (MA, KF). Any disagreements were resolved by discussion between the reviewers and consultation with a third researcher (BW) where needed.

For articles selected by both reviewers’ full texts were retrieved and once more screened for eligibility.

### Data extraction

A standardized and piloted extraction form was used to extract the data from the original studies by two reviewers independently (MA & KSF). Inconsistences in data extraction were resolved by discussion between the reviewers. The following information was extracted from each included study: the sample size, the study design, the country, methodology for measuring mortality, RR or OD with corresponding 95% CI, and population demographics where available. For country-wide studies population size was acquired from their respective government websites in the cases where it was not specified in the study.

### Quality assessments

Two reviewers (MA & KSF) assessed the methodological quality of all studies included by independently using the evaluation criteria for case-control and cohort studies as described by the Nine-Star Newcastle Ottawa Scale (NOS) [27]. Two reviewers independently ranked the quality of the studies included in the analysis according to NOS. The quality was assessed using three categories, selection, comparability and outcome. Disagreements were resolved by discussion between the reviewers. The quality was rated; low quality 0–4; moderate quality 5–6; and high quality 7–9.

### Statistical analysis

The measure of effect size was the RR with corresponding 95% CI. In studies where RR was not provided the data was converted according to; OR was converted into RR using the following formula: RR = OR/(1-P0) + (P0 x OR) with P0 being the incidence of the outcome in interest in the non-exposed group [28]. The standard error of approximate RR was calculated according to: SElog (RR) = SElog (OR) x log (RR)/log (OR), this method was also used to calculate CI by applying the formula to the upper and lower confidence interval of any adjusted odds ratio [29].

Heterogeneity was measured using Higgins & Thompson I^2^ [30]. For pooling effect size and the estimation of overall effect, a random-effects model approach was used. Standard error and Log of constructed RR was used to construct graphs for publication bias. Publication bias was evaluated by visual inspection of the funnel plot and Egger test for asymmetry. Due to the low number of studies we were unable to perform subgroup analysis of factors such as men to women ratio, nursing home status, prevention strategies, and national pandemic situation.

A value of *p* < 0.05 was considered statistically significant for all described analyses. Stata software version 14.0 (Stata Corp, College Station, TX) was used for all statistical analysis.

## Results

### Search results

**Fig. 1:**
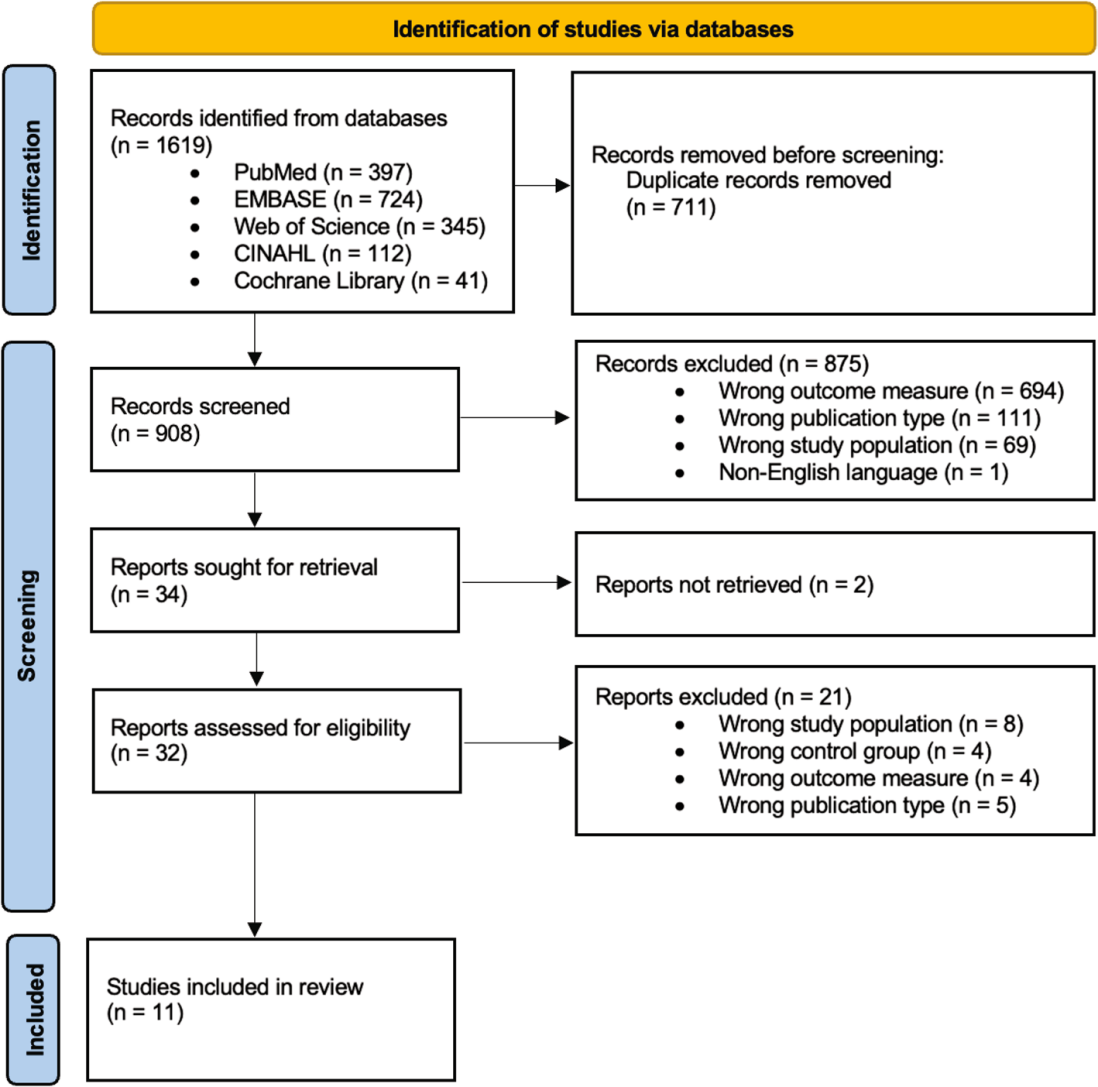
Flowchart of study identification and selection process as presented in a PRISMA 2020 diagram for new systematic reviews

The search strategy identified 1619 scientific articles within the relevant search criteria which were all retrieved. Deduplication screening via software and manual duplication checks provided 909 articles o be assessed for eligibility. After initial screening of title and abstract 32 articles were screened at full text. Eleven studies were included in the final analysis of which 10 were cohort studies and one was a case control study. The included studies were published during the time between January 1st, 2020 and March 31st, 2022. The PRISMA flow diagram illustrate the selection process of the included studies (Fig. 1).

### Study characteristics and study quality

A total of 11 published studies were included [17, 31–40]. The majority of studies were cohort studies. The geographical location of the studies included four studies from the US [36–39], three from the UK [31, 32, 40], two from Italy [33, 34], one from Norway [35] and one from Sweden [17]. Six studies used nation-level population data [17, 33, 35, 36, 38, 39], two studies used primary care data [31, 40], one study included data from an insurance registry [37], and two studies focused on smaller regional death registries [32, 34]. Studies varied in the time periods examined, only four studies consisted of data analysis of deaths stretching a time period of at least a year [17, 32, 38, 40]. Six studies focused on the first half of 2020 during the time when the world was experiencing the first wave of the COVID-19 pandemic [31, 34–36]. One study focused only on deaths occurring during December 2020 [37].

Full descriptive study characteristics including authorship, study design, the sample population, the control population, the aim, methodology, and assessment used in outcome was compiled from each study (Supplementary Table 1).

Quality of the studies according to NOS was mixed with 3 studies out of 11 ranking high, 5 were moderate and 3 were low (Table 1).

### Results of meta-analysis

**Fig. 2:**
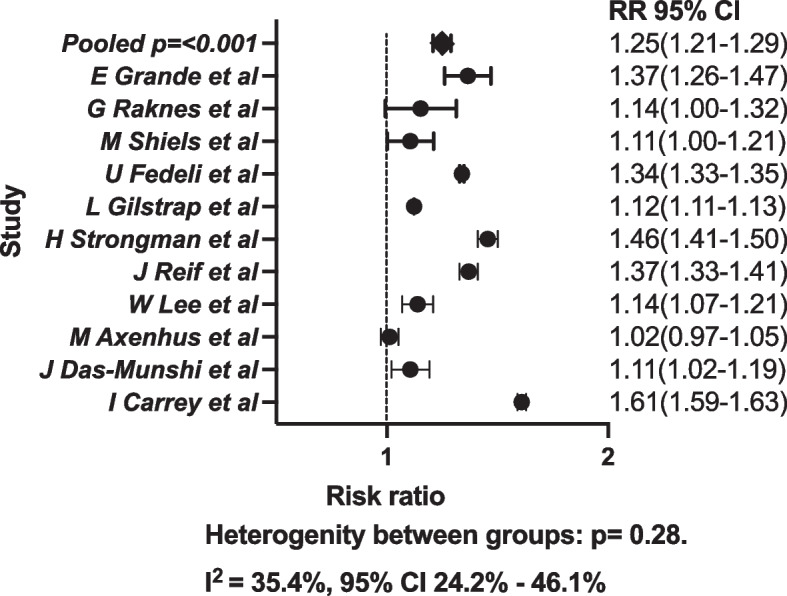
Forrest plot of the relative risk from each study and population with weighted pooled risk ratios. During the pandemic the risk of dying in dementia was increased compared to the pre-pandemic control period

We extracted or calculated equivalent RR from 11 original studies. The effect size of the studies was weighted according to the various study populations. When pooled, the results showed that mortality in dementia during the COVID-19 amongst people with dementia without confirmed COVID-19 was 1.25 (RR) when compared to the period before the pandemic (Fig. 2).

In total, 10 studies showed that dementia mortality was elevated during the COVID-19 pandemic, 1 study did not show a difference, although seasonal changes in mortality pattern were reported [17]. Heterogeneity between studies were I^2^ = 35.4, 95% CI 23.2–36.1%, *p* = 0.28. We were unable to determine if the prevalence of mild or severe dementia had different impact on mortality since no study characterized study populations according to severity of dementia. Due to the low number of included studies we were also unable to perform subgroup analysis according to prevalence of nursing home residents, prevention strategies, national pandemic situation, and female to male ratio.

### Publication bias and sensitivity analyses

**Fig. 3:**
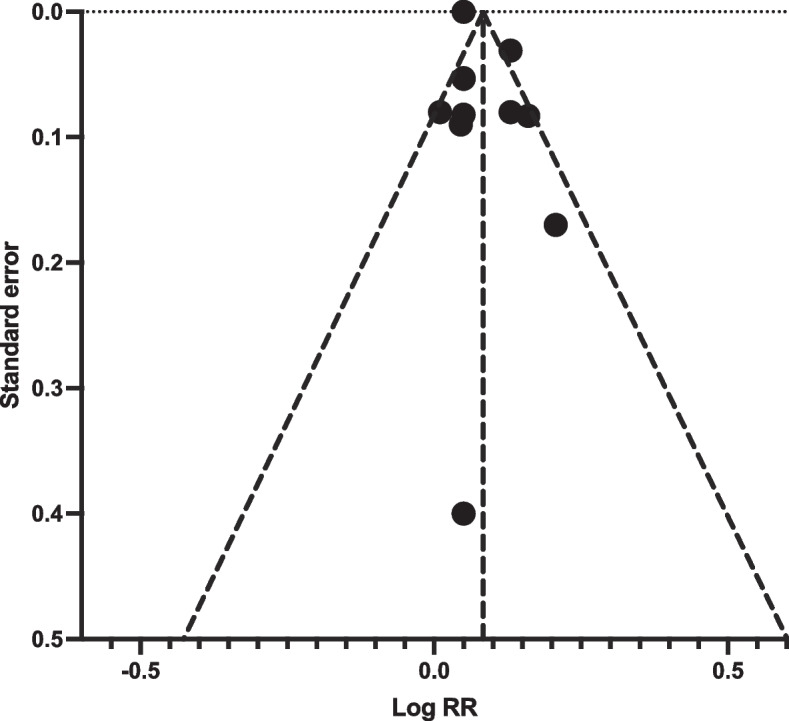
Funnel plot of included studies

Publication bias assessment was conducted by plotting a funnel plot using standard error and Log RR. Test of publication bias showed no significant. Egger’s test *p* = 0.612. Visual inspection of funnel plot shows no apparent bias at *P* > 0.05 (Fig. 3).

Sensitivity analysis was performed by removing one study at the time. Stepwise removal of studies did not produce a significant effect difference from the pooled results. This indicates that bias might be small or non-existent.

## Discussion

During the COVID-19 pandemic concern about care for the elderly and the vulnerability of the frail and elderly have been raised [11, 22]. It is well known that people with dementia have an increased mortality rate, compared to the general population [41]. This review highlights the changes in mortality of people with dementia without COVID-19 based on 11 studies from 5 countries assessing mortality amongst people with dementia during the COVID-19 pandemic.

The results of the meta-analysis indicate that the COVID-19 pandemic was associated with a higher risk of dying in people with dementia who do not have COVID-19. People with dementia experienced 25% higher risk of dying during the pandemic when compared to previous years.

There are several probable mechanisms which could increase the mortality amongst people with dementia during the pandemic. A redirection of care focus to acute COVID-19 related care might influence the care and resources dedicated to chronic and non-communicable diseases. Isolation has been shown to have a negative effect on the elderly and lack of social interaction is known to increase severity of symptoms amongst people with dementia [42]. Fewer opportunities to be physically active and engage in mental stimuli might also contribute to the physical deterioration of people with dementia [43]. The use of antipsychotics in people with dementia increased during the pandemic and these drugs are known to be associated with an increased mortality rate in people with dementia [44].

During the pandemic, studies have reported difficulties in supplying people living with dementia sufficient medical care. People living with dementia at care homes have experienced varying levels of social isolation and decrease in mental stimuli [45, 46]. Also home dwelling elderly with dementia and their care givers have reported loss of daily habits, loneliness and struggles to access regular health care [47–49]. There is also a difference between rural and urban areas with rural areas experiencing more COVID-19 related deaths than urban ones [50, 51]. Previous studies have implicated that people living with dementia have shorter lifespan and spend more time in rural nursing homes compared to their urban dwelling counterparts [52, 53]. The impact of this disparity in urban and rural mortality amongst people living with dementia, particular during the pandemic, is unclear but could be an interesting area of future research.

The death of an unusually large number of people with dementia could have significant impact on geriatric care around the world. Dementia disease burden might be wrongly quantified without proper assessment of dementia epidemiology in the aftermath of the pandemic. Changes in the incidence of dementia should therefore be monitored closely going forward, especially considering the concerns that the COVID-19 pandemic might precipitate a new “pandemic” of cognitive impairment [54, 55]. A recent study in COVID-19 survivors for example, found that cognitive decline was present even 1 year after infection, raising questions about the impact of COVID-19 infections the cognitive state of elderly populations [54].

The quality of the studies included in this review was on average good, and the risk of bias was considered small. We were unable to perform a subgroup analysis mainly due to the low number of studies included. A significant number of excluded studies focused on dementia as a risk factor in COVID-19 highlighting the fact that current research focus has been on establishing the risks associated with direct COVID-19 infections in people with dementia rather than studying all-cause dementia mortality rates.

The heterogeneity throughout the studies was small and pooled RR data did show an increased risk of death amongst people with dementia. Studies varied in the time frame during which dementia deaths were recorded and not all studies covered all of the pandemic waves in their respective countries. Yet almost all studies reported an increased risk of dying amongst people living with dementia. These findings suggest that pandemic waves were not primarily responsible for the increase in mortality rates and mortality increase is more likely due to systematic shortcomings such as decreased care or hospital overload.

This is the first review to focus on mortality amongst people living with dementia without COVID-19 during the COVID-19 pandemic and our findings could be influenced by several factors. Not all countries had the possibility to accurate attribute deaths due to COVID-19 during the beginning of the pandemic. Deaths amongst people with dementia is therefore difficult to accurately attribute. Neither were we able to find studies from China, India or other large populations from low-income countries. The dementia populations in these countries are significant and studies examining the mortality amongst these populations would be a valuable addition to our understanding of the risk which people with dementia has been exposed to worldwide during the COVID-19 pandemic. Lack of testing and inability to confirm COVID-19 diagnosis post-mortem might play a significant role in influencing mortality rates amongst people with dementia. Another limitation is that people with dementia can be categorized as not having COVID-19 due to non-systematic testing, atypical symptoms or false negatives.

This study describes the effects of the pandemic on people living with dementia in terms of mortality during pandemic conditions. This study suggests that preventing direct infections is not sufficient to adequately protect vulnerable populations during pandemic conditions. This data is important as it may help stakeholders in geriatric care to formulate future responses to pandemic care. Further studies should focus on the causation of increased mortality as this was left unanswered in this review. Variance between countries in terms of access to healthcare, prevention strategies, elderly care, and prevalence of people with dementia in care homes are factors that should be considered.

Many things can be done in order to address the rising mortality rates during a pandemic amongst people with dementia. Since the mortality of people with dementia might be connected to the general spread in society, reforms that address the spread should prove effective at mitigating mortality amongst people with dementia during pandemics.

Clinicians should be aware of the increased risk of mortality in people living with dementia. Although we were unable to prove this association in our study, nursing home clinicians should be aware of the possible connection between nursing home status and dementia mortality during pandemic conditions. Beside focusing on direct infections, clinicians should also promote actions that could be useful in lessening pandemic impact such as expanded testing of care personal, faster vaccine rollout and mask mandates [56–58]. Non-pharmacological interventions and new technologies can also help stakeholders to enable better support for people with dementia during times of uncertainty [59].

## Conclusion

Based on the existing body of literature, there has been a 25% increase in the risk of mortality amongst people living with dementia without COVID-19 during the pandemic. The reason for this increase is unclear but mortality amongst people living with dementia is likely associated with adjustable factors and could be mitigated by prevention strategies that limit pandemic impact on societal and healthcare functions. Awareness about this vulnerable population and tailored responses to new pandemic surges might help stakeholders limit the impact of future pandemic waves. Further research is warranted in order to identify mechanisms which drive mortality amongst people living with dementia during pandemics such as national prevention strategies and severity of dementia.

## Supplementary Information

**Additional file 1.**

**Additional file 2.**

## Abbreviations

AD: Alzheimer disease
COVID-19: Coronavirus disease 2019
NOS: Newcastle Ottawa Scale
OR: Odds ratio
PRISMA: Preferred Reporting Items for Systematic Reviews and Meta-Analysis
PROSPERO: International Prospective Register of Systematic reviews
RR: Risk ratio
SARS-CoV-2: Severe acute respiratory syndrome-coronavirus 2
